# The Individual Ictal Fingerprint: Combining Movement Measures With Ultra Long-Term Subcutaneous EEG in People With Epilepsy

**DOI:** 10.3389/fneur.2021.718329

**Published:** 2021-12-23

**Authors:** Troels W. Kjaer, Line S. Remvig, Asbjoern W. Helge, Jonas Duun-Henriksen

**Affiliations:** ^1^Center of Neurophysiology, Department of Neurology, Zealand University Hospital, Roskilde, Denmark; ^2^Department of Clinical Medicine, University of Copenhagen, Copenhagen, Denmark; ^3^Epilepsy Science, UNEEG medical A/S, Alleroed, Denmark

**Keywords:** subcutaneous EEG, accelerometry, EMG, seizure detection, ictal fingerprint, epilepsy

## Abstract

**Background:** Epileptic seizures are caused by abnormal brain wave hypersynchronization leading to a range of signs and symptoms. Tools for detecting seizures in everyday life typically focus on cardiac rhythm, electrodermal activity, or movement (EMG, accelerometry); however, these modalities are not very effective for non-motor seizures. Ultra long-term subcutaneous EEG-devices can detect the electrographic changes that do not depend on clinical changes. Nonetheless, this also means that it is not possible to assess whether a seizure is clinical or subclinical based on an EEG signal alone. Therefore, we combine EEG and movement-related modalities in this work. We focus on whether it is possible to define an individual “multimodal ictal fingerprint” which can be exploited in different epilepsy management purposes.

**Methods:** This study used ultra long-term data from an outpatient monitoring trial of persons with temporal lobe epilepsy obtained with a subcutaneous EEG recording system. Subcutaneous EEG, an EMG estimate and chest-mounted accelerometry were extracted from four persons showing more than 10 well-defined electrographic seizures each. Numerous features were computed from all three modalities. Based on these, the Gini impurity measure of a Random Forest classifier was used to select the most discriminative features for the ictal fingerprint. A total of 74 electrographic seizures were analyzed.

**Results:** The optimal individual ictal fingerprints included features extracted from all three tested modalities: an acceleration component; the power of the estimated EMG activity; and the relative power in the delta (0.5–4 Hz), low theta (4–6 Hz), and high theta (6–8 Hz) bands of the subcutaneous EEG. Multimodal ictal fingerprints were established for all persons, clustering seizures within persons, while separating seizures across persons.

**Conclusion:** The existence of multimodal ictal fingerprints illustrates the benefits of combining multiple modalities such as EEG, EMG, and accelerometry in future epilepsy management. Multimodal ictal fingerprints could be used by doctors to get a better understanding of the individual seizure semiology of people with epilepsy. Furthermore, the multimodal ictal fingerprint gives a better understanding of how seizures manifest simultaneously in different modalities. A knowledge that could be used to improve seizure acknowledgment when reviewing EEG without video.

## Introduction

People with epilepsy (PWE) experience repetitive, unexpected seizure episodes, caused by abnormal brain wave hypersynchronization leading to a range of signs and symptoms—the so-called semiology. Quantitative and qualitative characterization of semiology is the cornerstone in every case of epilepsy management. While seizure types tend to be similar from time to time within an individual PWE, many different seizure types exist in different PWE (1). Therefore, an objective description of the most common individual seizure characteristics for each PWE—the multimodal ictal fingerprint—could have multiple potential uses within epilepsy treatment (e.g., clinical management or seizure detection).

In epilepsy management, automatic device-based seizure detection may be useful for qualitative description of the seizure semiology in daily life. In the clinic, a concise and objective qualitative description of the seizure semiology can add value in the epilepsy diagnostic process for the healthcare professionals. With an ictal fingerprint available, the clinical management and treatment optimization might be less cumbersome. Including movement measures in the ictal fingerprint can potentially add clinical symptoms, which is an important part of the seizure semiology—both during the diagnostic process and treatment optimization.

In the outpatient setting, accurate seizure documentation remains an ongoing challenge. Seizure diaries, the current standard assessment of seizure counts at home, has been shown to be unreliable, potentially leading to over- or under-reporting (2). Both of which might lead to incorrect seizure treatment and complications for PWE (3). Furthermore, unobserved seizures have been associated with increased risk of morbidity and mortality through seizure related accidents, SUDEP etc. Hence, a seizure alarm making another person aware of the seizure may help reduce the risk (4). There exist several seizure alarms, of which most are based on movement (pressure, accelerometry, and EMG). However, focus on other modalities like ECG and EEG seems promising especially in seizures with a limited motor component (5). Many of the current alarm systems have a limited use due to low sensitivity, high false alarm rate, not being body worn or being obtrusive, hence not used (6).

For objective seizure counting and alarms, the need for improved combinations of devices and algorithms that work in everyday life settings is substantial. Novel subcutaneous EEG (sqEEG) devices present an intriguing hardware development which makes EEG recordings possible around the clock (3, 7–10). However, the use of these devices requires automatic algorithms to process the recordings due to the vast amount of data. Two possible approaches for improving these algorithms could be multimodal sensing and detection based on the individual ictal seizure semiology. Multimodal sensing is performed as standard in the epilepsy monitoring unit, but also examples of multimodal home monitoring are published (11). Combining sqEEG sensing with movement sensing is a simple way to capture both the electrographic and clinical motor parts of the seizure semiology during home monitoring. This approach is novel and exactly what is investigated in this study. Hereby the gap between home monitoring and the golden standard video-EEG in the epilepsy monitoring unit is narrowed.

This article presents a multimodal ictal fingerprint based on movement measures and ultra long-term sqEEG recorded in the everyday life of PWE. The aim is to explore if the concept of an ictal fingerprint can be used to describe and better understand the homogeneity of seizures within each PWE and the heterogeneity of seizures across PWEs. If that is the case, multimodal recordings could be used to supply doctors with an improved, subject-specific epilepsy semiology during outpatient monitoring. Furthermore, it could be used to improve seizure detection methods by increasing the knowledge of the heterogeneity of individual seizures.

## Materials and Methods

Data from an outpatient trial using ultra long-term minimally invasive sqEEG to monitor epileptic seizures constituted the basis for this work. For a detailed account on the study design, data collection procedures and demographics of the participating persons please refer to the initial report of the clinical trial (3).

### Study Population

To ensure a representative distribution of seizure data a minimum of 10 well-defined electrographic seizures were required for each participant. This meant that four out of nine participants were included in the present publication. All included persons suffered from medically refractory left temporal lobe epilepsy. Table 1 depicts person characteristics.

**Table 1:** Epilepsy and data characteristics for each person.

**Person ID**	**Ictal onset zone**	**Semiology**	**EEG data (h)**	Number of electrographic seizures (*N*)^*^
B	LT	FAS	1,552	25
E	LT	FIAS	1,147	15
G	LT	Uncertain	1,516	12
I	LT	FIAS	1,605	22

*FAS, focal aware seizure; FIAS, focal impaired awareness seizure; LT, left temporal*.

* *Number of seizures when the four FBTCS's (focal to bilateral tonic-clonic seizures) were excluded*.

In total, 78 electrographic seizures from the four persons (range: 12–25 seizures/person) were identified from the sqEEG data. All focal to bilateral tonic-clonic seizures (FBTCS) were excluded from the analysis (a total of four seizures from two different persons) to focus on defining the ictal fingerprint of seizures with a smaller motor component. These are the challenging ones to separate as opposed to FBTCS which can be detected using wearable devices (12), and in addition, they constitute most seizures. Thus, 74 electrographic non-FBTCS's constituted the basis for this work.

### Data Collection and Characteristics

**Figure 1:**
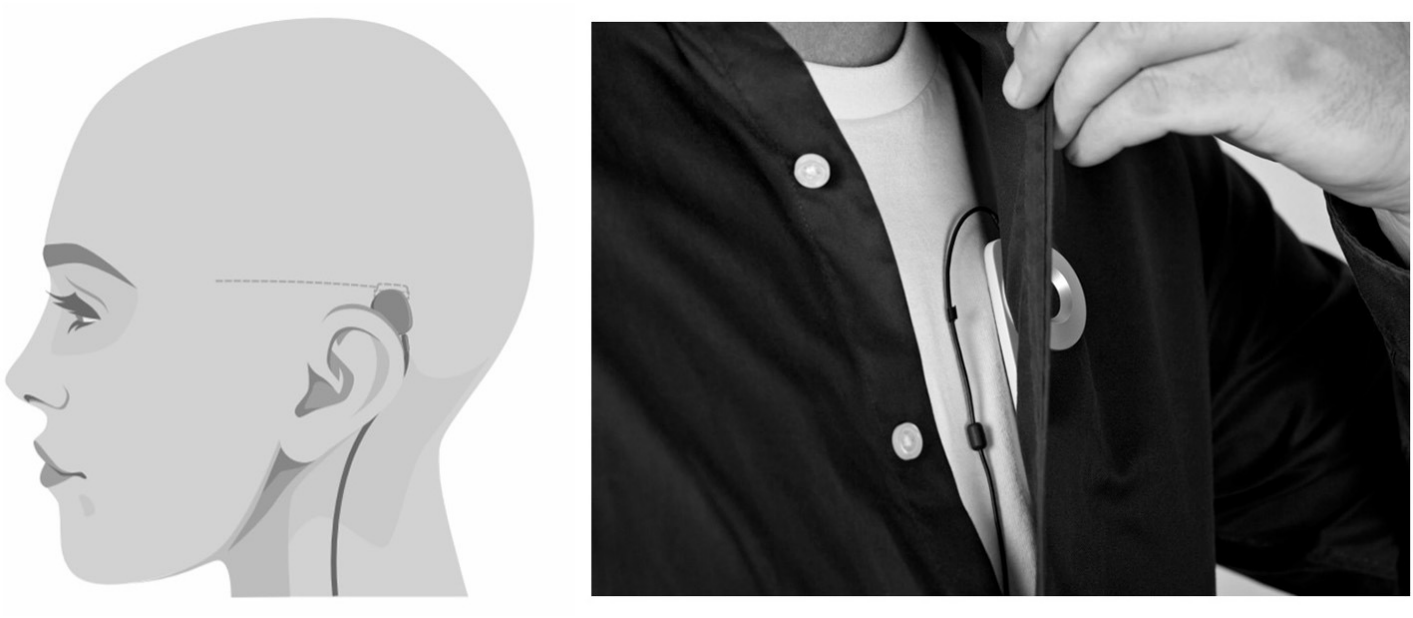
The SubQ solution and its placement in this study. *Left*: sagittal view of the head showing the placement of implant at the temporal region, recording 2-channel bipolar temporal sqEEG. *Right*: frontal view of the trunk demonstrating the placement of the external device, recording 3-axis accelerometry of the body trunk.

The sqEEG recorder system (24/7 EEG™ SubQ, UNEEG medical, Lynge, Denmark), referred to as the SubQ solution, consists of an implant and an external device. The implant consists of a 3-contact lead wire (yielding 2-channel bipolar EEG) and a small housing, implanted unilaterally under local anesthesia over the temporal region of interest (see Figure 1). The external device connects to the implant housing *via* an inductive link, powering the implant and recording/storing data (sampling rate: 207 Hz). The external device holds a 3-axis accelerometer (applied sampling rate of 10 or 20 Hz). As the external device is typically carried on the shirt as depicted in Figure 1, derivative accelerometer measures carry information on the orientation, posture, and movement of the body trunk.

Each person used the SubQ solution for 2–3 months of their everyday life. The total amount of outpatient sqEEG has been reviewed and labeled with “electrographic seizure” by a thorough review process of leading experts from three different institutions (in preparation for publication).

### Data Analysis

Of the large amounts of recorded data, ictal, pre-ictal and baseline data was extracted and applied in this study. All electrographic seizures were labeled with seizure onset and duration, defining the ictal period. The baseline and pre-ictal periods were extracted for comparison with the ictal periods to demonstrate that the activity during the ictal periods were different from the remaining signal. The baseline period was defined as a 1-min period ending 5 min before the seizure onset and the pre-ictal period was defined as the minute period preceding the seizure onset. A length of 1 min was deemed sufficient to minimize the influence of inherent signal variance. The first two and the last 2 s of the ictal periods and the last 2 s of the pre-ictal periods were excluded from the analysis to avoid transition phenomena.

In addition to the sqEEG and 3-axis accelerometry, an EMG signal estimate was extracted from the sqEEG, based on the frequency content above 20 Hz. The recording electrodes of the implant span the temporalis muscle, thus, recorded activity above 20 Hz is very likely temporalis activity (13).

To assess a multimodal ictal fingerprint across persons, ~70 features spanning all three modalities were calculated for the ictal and pre-ictal periods. To find a compact ictal fingerprint, it was decided to remove the redundant features. For this purpose, a Random Forest classifier was trained in a 5-fold cross-validation scheme with nine different hyperparameter settings (14). The hyperparameter settings were a grid search over the number of trees (25, 50, 100) and the minimum number of samples to split a node (2, 4, 6). The remaining hyperparameters were the default parameters used by the Random Forest Classifier function of the python package sklearn (v. 0.24.1). The classification task consisted of separating ictal periods between persons. From the best performing model, a feature importance parameter was extracted based on the internal gini impurity measure, which determines the node splits in each of the decision trees. The five most discriminative features were selected for further analysis (hereafter referred to as the reduced feature space).

A principal component analysis was performed in the reduced feature space to visualize the person-specific, feature-based clusters of seizures. A seizure centroid was computed for each person in the space spanned by the principal components by averaging over all seizures. Then each seizure was assigned to the person with the centroid which was closest measured using Euclidean distance. The accuracy of this simple clustering was calculated.

To illustrate the uniqueness of the multivariate ictal fingerprint, radar charts of the ictal feature medians and interquartile ranges were displayed for each person. All features were Z-score normalized for optimized visualization and comparison.

To demonstrate that the ictal periods differ from the baseline and pre-ictal periods within persons, a distance-to-ictal-cluster-average vs. distance-to-preictal-cluster-average plot was made for all ictal and pre-ictal periods. Distances were to person-specific cluster averages, calculated as Euclidean norms in the reduced feature space.

## Results

### Feature Space Reduction

The reduced feature space included one accelerometer-based feature: the x-axis accelerometer component; one estimated EMG feature: the >20 Hz power at the proximal electrode contact point; and three EEG-based features: the relative power in the delta (0.5–4 Hz), low theta (4–6 Hz), and high theta (6–8 Hz) band.

### Ictal Clustering

**Figure 2:**
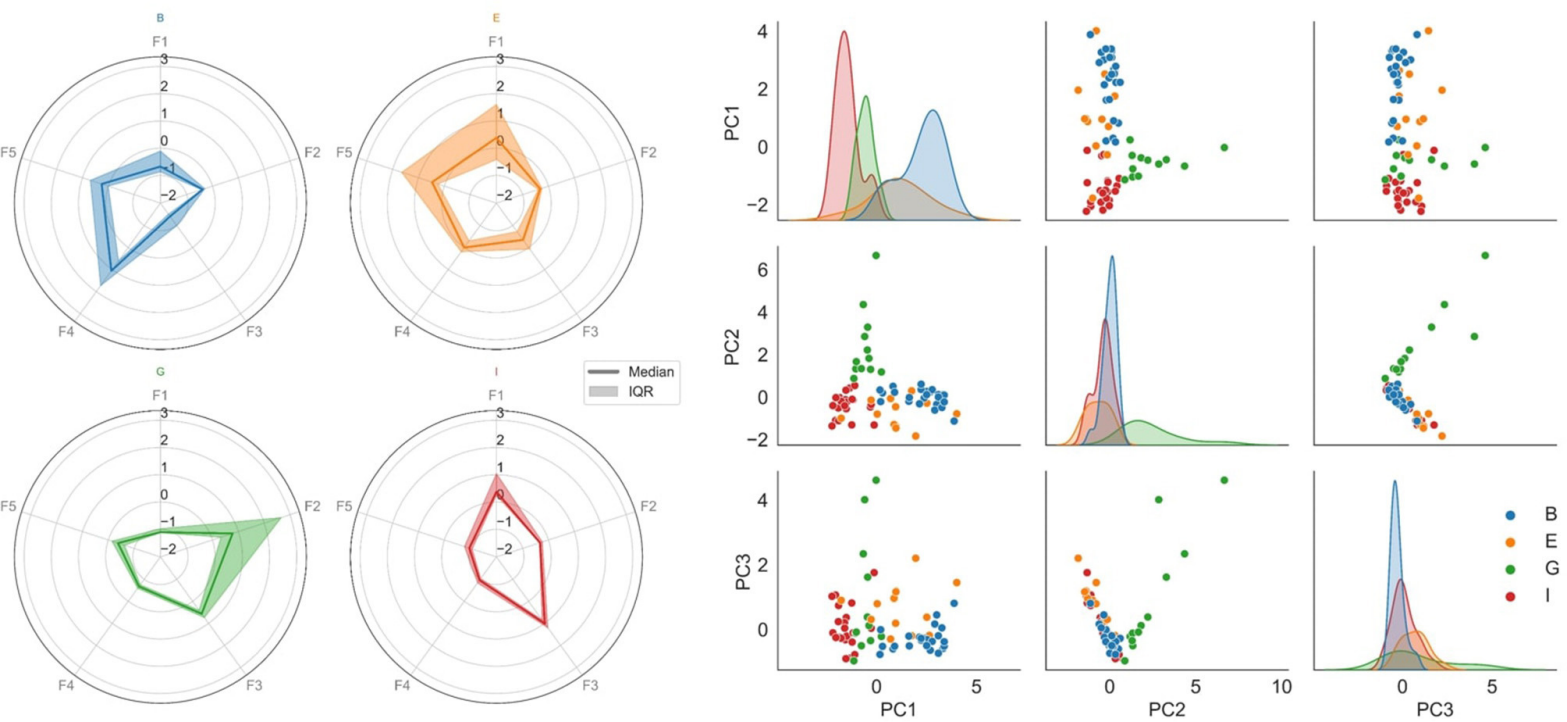
The multimodal ictal fingerprint. Each color represents a separate participant (blue, orange, green, and red are person B, E, G, and I, respectively). *Left:* Radar charts of the ictal feature medians and interquartile ranges for each person. F1: X-axis acceleration component; F2: EMG power proximal; F3: EEG relative delta power; F4: EEG relative low theta power; F5: EEG relative high theta power. The heterogeneity across persons is shown. The ictal fingerprint of person B (blue) is dominated by EEG theta power elevation. The fingerprint of person E (orange) has a partial overlap with elevated theta activity. However, the movement component (F1) is a major contributor for this fingerprint. The movement component is also substantial for person I (red), but with no remarkable theta elevation. The ictal fingerprint of person G (green) is not dominated by either of the mentioned features; instead, elevated EMG activity is the main contributor. *Right:* Pair plot of the first three principal components of the reduced feature space of the ictal periods, where each dot represents a seizure. Scatterplots are shown for each pairing of the principal components, and marginal distributions are plotted along the diagonal (layered kernel density estimates). Within-person clustering and separation across persons are shown. The seizures of each individual could be separated from the rest with an 84.5% accuracy.

The pair plot of the first three principal components of the reduced feature vectors (right chart of Figure 2) shows that the seizures group together in person-specific, feature-based clusters. Using a simple clustering method, the accuracy of separating the seizures of all subjects reached 84.5%. By visual inspection of the figure, it can be observed that B, G, and I were more distinguishable than E and separating only B, G, and I could be done with an accuracy of 93.1%. To some extent, the feature characteristics of person E seem to group with person I. According to Table 1, their seizure semiologies are alike, both experiencing focal impaired awareness seizures.

### The Ictal Fingerprint

In the left graph of Figure 2, radar plots of the ictal feature medians and interquartile ranges demonstrate the heterogeneity across persons. E.g., the ictal period of person G is dominated by high EMG activity, whereas it is not the case for any other person. Likewise, the relative low theta power is elevated for the ictal periods of person B, whereas this does not dominate the ictal fingerprints of the remaining persons.

### Baseline and Pre-ictal to Ictal Separation

**Figure 3:**
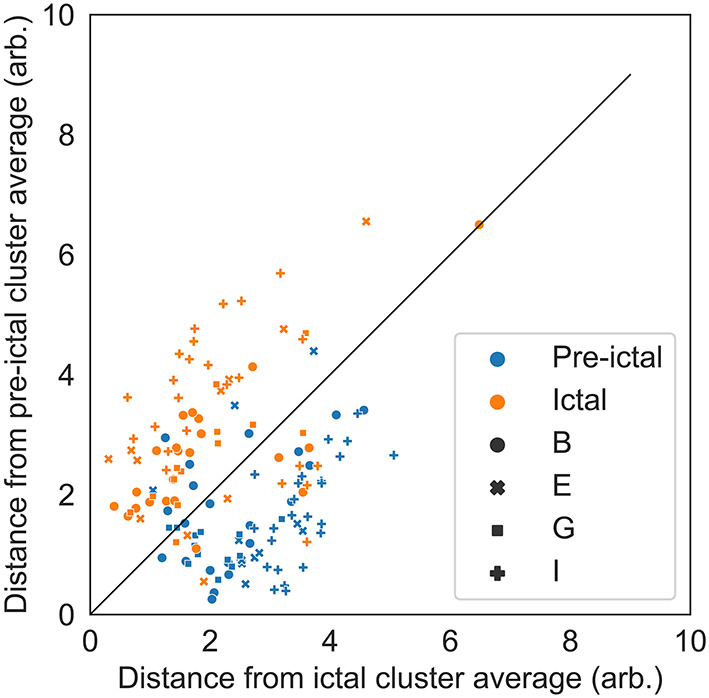
Distance-to-ictal-cluster-average vs. distance-to-preictal- cluster-average for all ictal and pre-ictal periods to illustrate the separation capability. Distances are to person-specific cluster averages, calculated as Euclidean norms in the reduced feature space. The solid black line represents the class separation. The separation accuracy was 83.8%. Period is shown with different colors (blue are pre-ictal periods and orange are ictal periods) and the subject ID is shown with different marker types (circles, crosses, squares and plusses are persons B, E, G, and I, respectively).

Figure 3 includes the pre-ictal periods to show pre-ictal to ictal separation and Table 2 present the statistics of the separation task. The cluster center distances show a separation accuracy of 83.8% for the pre-ictal to ictal separation and 88.1% for the baseline to ictal separation. Thereby, it is indicated that the created ictal fingerprints are truly ictal, and not general person-specific multimodal fingerprints.

**Table 2:** Accuracy, sensitivity, specificity, positive predictive value, and negative predictive value of separating ictal from pre-ictal periods.

**Statistic**	**Value (%)**	**Confidence interval (%)**
Accuracy	83.8	±6.1
Sensitivity	80.3	±6.5
Specificity	87.3	±5.5
Positive predictive value	86.4	±5.6
Negative predictive value	81.6	±6.4

## Discussion

Seizures can be split up into separate seizure types and the same seizure type can manifest differently for each patient (15). Here we have demonstrated that the concept of an ictal fingerprint is meaningful when based on data from three different modalities: EEG, EMG, and accelerometry all recorded with the SubQ solution in four different PWE. We have not managed to find previous efforts showing this phenomenon even though previous studies have tried to use multiple modalities in seizure detection algorithms (16). For that reason, the purpose was not to achieve the highest possible separation of seizures in the person-specific, feature-based clusters. Instead, it was to demonstrate that an ictal fingerprint exists defined by easily interpretable features and discuss the advantages of using multiple modalities. Collecting data on individual seizures from multiple modalities has the potential to improve clinical treatment management. The proposed ictal fingerprint supplies information that could give healthcare professionals in the clinic a more detailed description of any specific individual's seizure semiology. Part of this information would allow to distinguish between clinical and subclinical seizures. A task that is not possible with unimodal data because EEG is needed to discover the subclinical seizures and other modalities, such as EMG or accelerometry, are needed to determine whether a seizure is clinical.

PWE need a solution for seizure detection that works in their everyday life, and multiple unimodal setups have been proposed. The performance of EEG based alarms has so far been problematic, potentially challenging clinical utility and user tolerance (17, 18). Most of the studies regarding EEG-based alarms show only moderate sensitivity, tolerable false detection rates and are performed on data obtained under standardized circumstances, e.g., in the hospital and instead of during everyday activities (19). Most commercially available alarms are triggered by movement (accelerometry, EMG), or sympathetic activity (ECG, electrodermal response), requiring either a significant motor component or autonomic component of the seizures to work (20). Often movements, exercise or change in autonomic load cause false detections.

The crucial challenge when designing devices and algorithms for seizure detection is to detect all true seizures while avoiding false alarms, i.e., obtaining high sensitivity and specificity. Achieving this goal requires data in which seizures are separable from background activity. Our findings in four persons with temporal lobe epilepsy show that the modalities which describe the seizures best are different from person to person. Visual inspection makes it clear that the seizures can be grouped into person-specific, feature-based clusters, meaning they are generally more similar within PWEs and more different between PWEs (Figure 2).

EEG signals differ from person to person to a degree where EEG has even been proposed as a modality that could be used for biometric recognition (21–23). It was therefore expected that the persons could be distinguished based on multiple modalities incl. EEG. Figure 3 illustrates that there is not only a multimodal overall fingerprint but also a separate ictal fingerprint as the pre-ictal periods could be separated from the ictal periods.

This study only presents the ictal fingerprint of four PWEs. It is therefore unknown to what degree the proposed ictal fingerprint will overlap between many individuals. While the reduced feature space represents commonly used features from the selected modalities, the proposed ictal fingerprint contains more features than there are PWEs. Separability of the person-specific, feature-based clusters presented in Figure 2 would be expected to decrease with increasing number of persons. This could lead to a need for a revised model of the ictal fingerprint incorporating other and possibly more features. The approach described in this paper is novel in the way it combines movement and EEG in an everyday life setting. What is also special, is that the accelerometer used in this study is placed on the chest revealing movement of the trunk rather than extremities, which is usually the case for seizure detectors. Finding that body trunk movements can contribute to the ictal fingerprint might be surprising. However, it is advantageous that measurement devices placed on extremities are not required in order to limit the number of devices to be worn by the PWEs.

Introducing the concept of an ictal fingerprint has the potential to improve the PWE's knowledge of their own seizures which might increase their device compliance. The readiness of PWEs to use wearables in everyday life requires that individual needs are addressed, and expectations are met to better understand their life situation. A device can be perceived by the PWEs as a lifeline to health and access to healthcare professionals (24).

In summary, our findings in four persons with temporal lope epilepsy show that it is possible to create unique individual ictal fingerprints, where the multimodal characteristics describing the ictal periods best, differ from person to person while staying consistent within each person. Individual ictal fingerprints may enhance clinical management, improve seizure acknowledgment and detection algorithms, and lead to better personal healthcare experiences.

## Data Availability Statement

The data analyzed in this study is subject to the following licenses/restrictions: the data consists of huge amounts of raw EEG, which are not available to the public. The study where the data was collected is registered in ClinicalTrials.gov (NCT02946151). Requests to access these datasets should be directed to Troels W. Kjaer, twk@regionsjaelland.dk.

## Ethics Statement

The studies involving human participants were reviewed and approved by the Committee of Science Ethics for Region Zealand (SJ-551). The patients/participants provided their written informed consent to participate in this study.

## Author Contributions

JDH and TK contributed to conception and design of the study where the applied data has been recorded. AH and LR performed the statistical analysis. AH, LR, and TK wrote sections of the manuscript. All authors contributed to manuscript revision, read, and approved the submitted version.

## Conflict of Interest

TK consults for UNEEG medical A/S. LR, AH, and JDH are employees of UNEEG medical A/S.

## Publisher's Note

All claims expressed in this article are solely those of the authors and do not necessarily represent those of their affiliated organizations, or those of the publisher, the editors and the reviewers. Any product that may be evaluated in this article, or claim that may be made by its manufacturer, is not guaranteed or endorsed by the publisher.
